# Comparing machine learning techniques for neonatal mortality prediction: insights from a modeling competition

**DOI:** 10.1038/s41390-024-03773-5

**Published:** 2024-12-16

**Authors:** Brynne A. Sullivan, Alvaro G. Moreira, Ryan M. McAdams, Lindsey A. Knake, Ameena Husain, Jiaxing Qiu, Avinash Mudireddy, Abrar Majeedi, Wissam Shalish, Douglas E. Lake, Zachary A. Vesoulis

**Affiliations:** 1 University of Virginia, Department of Pediatrics, Division of Neonatology, Charlottesville, VA USA; 2 University of Texas Health San Antonio, Department of Pediatrics, Division of Neonatology, San Antonio, TX USA; 3 University of Wisconsin-Madison, Department of Biostatistics and Medical Informatics, Madison, WI USA; 4 University of Wisconsin-Madison, Department of Pediatrics, Division of Neonatology, Madison, WI USA; 5 University of Iowa, Department of Pediatrics, Division of Neonatology, Iowa City, IA USA; 6 University of Utah, Department of Pediatrics, Division of Neonatology, Salt Lake City, UT USA; 7 University of Iowa, Iowa Initiative for Artificial Intelligence, Iowa City, IA USA; 8 Research Institute of the McGill University Health Center, Montreal Children’s Hospital, Department of Pediatrics, Division of Neonatology, Montreal, Canada; 9 Washington University in St. Louis, Department of Pediatrics, Division of Newborn Medicine, St. Louis, MO USA

## Abstract

**Background:**

Predicting mortality risk in neonatal intensive care units (NICUs) is challenging due to complex, variable clinical and physiological data. Machine learning (ML) offers potential for more accurate risk stratification.

**Objective:**

To compare the performance of various ML models in predicting NICU mortality using a team-based modeling competition.

**Methods:**

We conducted a modeling competition with five neonatologist-led teams applying ML techniques—logistic regression, CatBoost, neural networks, random forest, and XGBoost—to a shared dataset from over 6,000 NICU admissions. The dataset included static demographic and clinical variables, alongside daily samples of heart rate and oxygen saturation. Each team developed models to predict mortality risk at baseline and within 7 days. Models were evaluated using the area under the receiver operator characteristic curve (AUC). Results were presented at a national meeting, where an audience poll ranked models before AUC results were revealed.

**Results:**

The audience favored the most complex model (CNN) for real-world application, though logistic regression achieved the highest AUC on test data. Teams employed varied feature selection, tuning, and evaluation strategies.

**Conclusions:**

Logistic regression outperformed more complex models, highlighting the importance of selecting modeling methods based on data characteristics, interpretability, and expertise rather than model complexity alone.

**Impact:**

- By demonstrating that model complexity does not necessarily equate to better predictive performance, this research encourages the careful selection of modeling approaches.

## Introduction

Early identification of infants at high mortality risk is essential for timely intervention, risk stratification, and improved outcomes. However, accurate mortality risk stratification of neonatal intensive care unit (NICU) patients continues to challenge clinicians, as it involves interpreting a wide array of static and dynamic clinical data, which have inconsistent correlations with pathology.^1^ In recent years, artificial intelligence (AI) methods, particularly machine learning (ML) models, have shown promise in overcoming these barriers and providing improved risk stratification and clinical outcome prediction^2–4^ in critical care settings.^2^ Despite the progress, there is limited evidence directly comparing the performance of various ML approaches in predicting neonatal mortality.^2,5^

To showcase the opportunities and challenges of using ML to solve clinical problems, we challenged neonatology physician-scientists with data science and ML expertise to participate in a modeling competition held during the annual Pediatric Academic Societies Meeting, a national pediatric research conference. Five teams were tasked with applying different ML modeling approaches to the same dataset, aiming to predict neonatal mortality. The competition format allowed for a unique, side-by-side comparison of various ML approaches, with each being evaluated using the same dataset, methods, and performance metrics.

Derived from anonymized, real patients, the dataset included baseline demographic and clinical variables alongside daily 10-minute records of heart rate (HR) and oxygen saturation (SpO_2_) data sampled every two seconds.^6^ The large, multi-modal dataset allowed participants to explore ML approaches using both static clinical risk factors and dynamic physiologic data into prediction models. The competition included two tasks: (A) predicting baseline NICU mortality risk using variables known at birth and (B) daily predictions of death within seven days using combined clinical and physiologic data.

## Methods

### Dataset and competition set-up

**Fig. 1:**
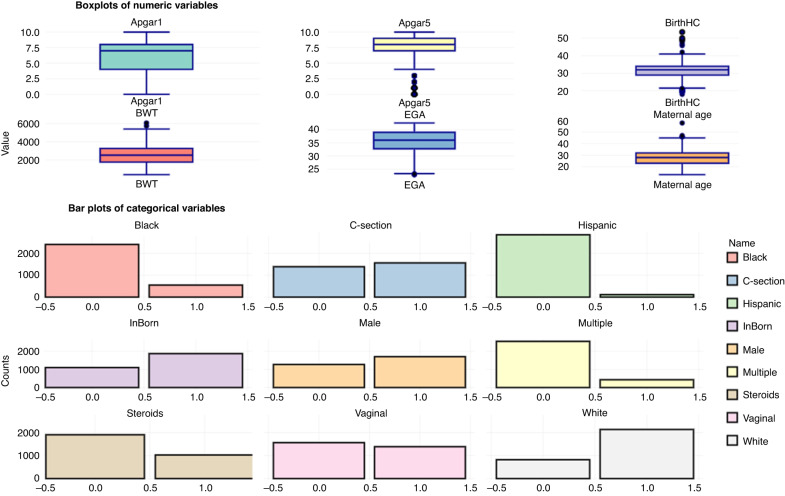
Summary plots of clinical variables using an online data exploration application. Numeric variables are displayed as box and whisker plots (top panel) and categorical variables are shown as bar plots (bottom panel). The plots were generated using an open-source application developed by Team “Texas Data Wranglers”, available online at https://neomindai.com/.

We previously published a de-identified dataset^6^ for model training, which included 2965 infants admitted to a single NICU over a 5-year period. A separate dataset of 2999 infants, admitted to the same NICU over a later 5-year period, was used for model testing. Infants of any birth weight or gestational age were included. Table 1 shows the summary statistics of the clinical variables included in the data sets used by all teams for training and testing. Figure 1 shows the distributions of these variables, accessible via an online tool at neomindai.com.

**Table 1:** Cohort Characteristics and missing data rates, grouped bgy data sets used for model training and model testing.

Variable	Training dataset (*N* = 2964)		Test dataset (*N* = 2999)	
Variable	Summary statistic, mean ± SD or N (%)	N (%) with the variable missing	Summary statistic, mean ± SD or N (%)	N (%) with the variable missing
**GA (weeks)**	35.2 ± 4.5	0	34.7 ± 4.5	0
**Birth Weight (grams)**	2495.1 ± 995.1	2 (0.06%)	2423.9 ± 1005.8	8 (0.2%)
**Maternal Age (years)**	28.08 ± 6.1	230 (7.7%)	28.02 ± 6	237 (7.9%)
**Birth Head Circumference (cm)**	31.3 ± 3.9	118 (3.9%)	31.1 ± 4	344 (11.4%)
**Male sex**	1697 (57%)	0	1713 (57.1%)	0
**Race/Ethnicity**		7 (0.2%)		116 (3.8%)
**Black**	552 (19%)		568 (19%)	
**White**	2135 (72%)		2030 (67%)	
**Other**	278 (9%)		316 (11%)	
**Hispanic**	113 (4%)		198 (6%)	
**Outborn birth**	1102 (37%)	0	1135 (38%)	0
**Cesarean delivery**	1564 (53%)	12 (0.4%)	1575 (53%)	129 (4.3%)
**Antenatal steroid exposure**	1030 (35%)	31 (1.05%)	855 (28%)	1100 (36.6%)
**1-minute Apgar**	6 ± 2.6	32 (1.07%)	6 ± 2.6	48 (1.6%)
**5-minute Apgar**	7 ± 2	33 (1.1%)	7 ± 2	50 (1.6%)

In addition to baseline clinical and demographic variables, the datasets contained daily 10-minute random samples of continuous heart rate (HR) and oxygen saturation (SpO_2_) data. Continuous HR and SpO_2_ monitoring data, sampled at 0.5 Hz, were routinely archived for all patients from NICU admission to discharge over the ten-year period. Thus, the dataset included daily measurements of 300 HR and 300 SpO_2_ values for each NICU patient and for every patient-day. There were 65,354 daily 10-min HR and SpO₂ values in the training dataset and 61,571 daily 10-min HR and SpO₂ values int the test dataset. Eight features were calculated from the daily 10-minute HR and SpO₂ raw data, including the mean, standard deviation, minimum, and maximum values. Teams were permitted to engineer new calculated features from the raw data.

The competition tasked teams with developing two types of models: (1) a baseline model using any combination of demographic and clinical variables to predict death before NICU discharge, and (2) a dynamic model that incorporated HR and SpO_2_ data to make daily predictions of death within seven days. The goal of the baseline models was to demonstrate a traditional approach to prediction, using static clinical variables at a single point in time. Conversely, the dynamic models aimed to demonstrate the additional insights provided by physiologic data, testing each model’s capability to make predictions based on repeated measures. The dynamic prediction model has clinical utility by offering early warning signals using information from vital sign patterns, which are not readily discernible in standard clinical practice.

The test data set was published without mortality outcomes so that teams could take their models developed using the training data set and generate predicted probabilities on the test data set while remaining blinded to outcomes. Each team submitted their test data predictions to independent moderators, who were unblinded to outcomes allowing them to calculate performance metrics for each team’s model. On the day of the conference, each team was given the opportunity to convince the audience of their model’s superiority using training data performance and strengths of their methods. Teams did not know the results of model validation before their presentations.

At the conclusion of the session, the independent moderators revealed model validation performance in the form of the area under the receiver operator characteristic curves (AUC) of each team’s model along with the audience voting results.

### Modeling teams and their methods

Five clinical neonatologists with ML research experience were selected and assigned different modeling methodologies to apply to a common data set and prediction task. They were allowed to form teams with data science collaborators and given five months to develop their models. Each team submitted its final model and predictions on a held-out test dataset to the organizing moderators, who then calculated and ranked the models based on their AUC.

The methods used by each team are outlined in the sections below. For each team and model, we briefly describe the general methodology^7^, steps used for data exploration, feature engineering, training the model, and evaluating the model’s performance in the training data set.

### Team “Wahoo Predictors”: Logistic Regression methods

The team from University of Virginia used a multivariable logistic regression model. Multivariable logistic regression models the relationship between multiple independent variables and a binary dependent outcome, coded as 0 or 1. This technique extends simple logistic regression by allowing the inclusion of multiple predictors, which can be continuous, categorical, or mixed. The model estimates the probability of the outcome occurring as a function of the independent variables, using the logistic function to ensure that the predicted probabilities fall between 0 and 1. The coefficients produced by the model represent the log odds of the outcome associated with each predictor, adjusted for the presence of other variables in the model. Multivariable logistic regression is particularly useful for its explainability, making it a helpful methodology when understanding the influence of multiple factors on a binary outcome is critical.

They analyzed predictors using univariate regression to evaluate the strength and shape of the association of each predictor with mortality, looking for non-linear relationships. Restricted cubic splines with up to 3 knots were incorporated for variables with non-linear relationships. They used Ridge regression for feature selection, removing highly correlated variables and weak predictors. Using their prior research experience with time series analytics, they engineered a novel HR feature to detect very low HR variability. This feature was calculated from the daily 10-min raw HR data as the probability of successive HR increases. When this probability is low, the HR variability is low, which this team has studied extensively as a signature of neonatal illness.^8,9^

The team trained their baseline mortality risk model using four clinical variables (5-minute Apgar score, gestational age, birth head circumference, and multiple gestation), of which two included a non-linear term (gestational age and birth head circumference). Next, they trained a daily model to predict death within seven days. This model included four HR/SpO_2_ features (HR increases, SpO_2_ max, HR max, SpO_2_ mean) and three non-linear terms. They used 10-fold cross-validation during training to optimize the performance without overfitting. In addition, they used a robust covariance matrix method to account for repeated measures.

### Team “Baby Badger”: CatBoost methods

The team from University of Wisconsin-Madison used a method called CatBoost, which uses an algorithm for gradient boosting on decision trees. This method excels in processing complex, varied data and can perform well with small datasets. CatBoost models are easy to implement, which can help to streamline deployment in the clinical setting. Other advantages include quick processing and training time and a reduced risk of overfitting through the use of ordered boosting.

To identify the most important predictors, they utilized SHapley Additive exPlanations (SHAP) values.^10^ From these results, they selected six features: HR standard deviation, SpO_2_ maximum, SpO_2_ mean, 5-minute Apgar score, multiple birth, and post menstrual age. They implemented 10-fold cross-validation to optimize hyperparameters and performance. Model performance was evaluated using cross-validated AUC and F1 scores. After tuning model parameters and final feature selection, the model was trained using the entire training dataset without cross-validation or split-sample validation.

### Team “Lady Hawks”: Neural Network methods

The team from the University of Iowa employed a hybrid approach involving different methods for modeling time-series data (HR and SpO_2_) and the baseline clinical parameters. The preprocessing pipeline was tailored to handle these two data types separately, ensuring that each type of input was optimally prepared for the predictive model.

For the baseline clinical parameters, Team “Lady Hawks” first performed feature selection using a Random Forest model to identify the most important features. This step helped reduce dimensionality and focused the model on the most relevant clinical variables.

For the time-series data (HR and SpO_2_), a convolutional neural network (CNN) autoencoder was used to compress the data. The CNN autoencoder extracted low-dimensional features from the high-frequency time-series input by learning efficient representations through a combination of convolutional encoding and decoding layers. This reduced feature set was designed to capture the essential patterns and trends in the data while discarding noise. The compressed features from the CNN autoencoder and the selected clinical variables from random forest were then combined into a single feature set, which served as the input to an artificial neural network (ANN).

After splitting the data set into training (80%) and validation (20%) sets, the training data was up-sampled to balance the minority class (death events). This was done to avoid bias towards the majority class (survival) as the model learned the associations of patterns in the data with the outcome.

### Team “Mountain Babies”: Random Forest methods

The team from the University of Utah used a modeling method called Random Forest. Random Forest is a method used for classification or regression tasks that operates by constructing many decision trees during training and outputting the mode of the classes (classification) or the mean prediction (regression) of the individual trees. It improves predictive accuracy and controls overfitting by averaging multiple decision trees created using random subsets of the training data and random subsets of features for each split in the trees. This randomness introduces diversity among the trees, leading to a more robust and generalizable model that is less sensitive to the noise in the data.

Starting with all 28 variables and default parameters, they tuned the model by adjusting parameters and comparing the out-of-bag (OOB) estimates of error rate. They found poor predictive performance with an OOB estimate of an error rate of 0.41% and addressed this by duplicating the minority class in the outcome (death), similar to the approach used by the Iowa team.

They evaluated feature importance using the mean decrease in the Gini Index and identified PMA, birthweight, age, birth head circumference, HR standard deviation, and 5-minute Apgar as the top predictors. They also evaluated the partial dependence of variables to evaluate feature performance at a granular level. After building a second, optimized model using tuned model parameters and selected features as predictors, they retrained the model, evaluating performance on the training data set using a confusion matrix to examine accuracy.

### Team “Texas Data Wranglers”: XGBoost methods

XGBoost (Extreme Gradient Boosting) is a powerful, efficient, and scalable ML algorithm that implements the gradient boosting framework for both classification and regression tasks. It builds an ensemble of decision trees in a sequential manner, where each tree corrects the errors of its predecessor by focusing on the residuals or errors made by the previous trees. XGBoost stands out for its high speed and performance, achieved through optimization techniques such as regularization to prevent overfitting, parallel processing to speed up computation, and advanced tree-pruning methods. These enhancements make XGBoost one of the top choices for ML tasks, particularly in scenarios where predictive accuracy is crucial. Disadvantages include the complexity of modeling with many hyperparameters and risk of overfitting if the tuning is not performed properly. Training an XGBoost model requires high computational power, especially with large datasets.

The team from University of Texas Health – San Antonio performed extensive data exploration using a custom-built automated data exploration tool to support feature engineering, now available at neomindai.com (Fig. 1). The primary objective was to predict death within 7 days, and predictions must be updated on a daily basis. To support this model need, lag features were created from the raw vital sign data, representing values of variables at a point in the recent past. For example, hr_mean_lag1 represents the heart rate mean from the previous day, hr_mean_lag2 from two days ago, and so on, up to 7 days (hr_mean_lag7). This daily lagging allowed the model to capture how the history of vital signs, such as heart rate variability and oxygen saturation, cumulatively influenced the likelihood of death within the next 7 days. By including lag features for multiple vital sign features (e.g., HR standard deviation, HR maximum, SpO_2_ mean, etc.), the model could incorporate the temporal trends and fluctuations that are crucial for predicting clinical deterioration.

After training the XGBoost model, a variable importance plot was generated to visualize the contribution of each feature to the model’s predictions. The importance of features was ranked based on their impact on model performance, and the top 10 most important features (4 demographic variables, 6 vital sign features) were selected for further analysis. These top features were expected to provide the most predictive power for identifying patients at high risk of death within the next 7 days. The model’s parameters included a learning rate of 0.1 and a maximum depth of 6. Team Texas used 10-fold cross-validation and AUC to evaluate the model and applied early stopping with a threshold of 10 rounds to ovoid overfitting their model and reduce computation times.

## Results

The modeling challenge attracted an audience of approximately 200 conference attendees, mainly comprised of mid-career neonatologists who reported themselves as having at least some understanding of ML methods but not expert-level knowledge.^11^ After the teams presented their modeling method, approach, and training dataset performance, electronic polling revealed audience perceptions on which model would perform best in real-world data (Table 2). Prior to the test data results, the neural network model was widely favored, while the logistic regression model was deemed the least likely to succeed. However, the final rankings based on test data AUC demonstrated the opposite: the logistic regression model achieved the highest AUC, outperforming all other models, including the more complex neural network. Table 2 shows the AUCs of each team’s baseline and combined model in the training and test datasets.

**Table 2:** Model evaluation using the area under the receiver operator characteristic curve (AUC) and audience response polling.

	Audience Poll Rank	Demo Train AUC	Demo Test AUC	Demo+VitalsTrain AUC	Demo+VitalsTest AUC	Final Rank
Wahoo Predictors	5	0.724	0.758	0.855	**0.818**	**1**
Baby Badger	3	0.737	0.769	0.837	**0.798**	**2**
Mountain Babies	4	0.861	0.730	1.00	**0.771**	**3**
Texas Data Wranglers	2	0.870	0.738	0.775	**0.725**	**4**
Lady Hawks	1	0.800	0.756	0.980	**0.652**	**5**

Teams presented their modeling methods and performance of their combined model (including both static demographic and clinical variables and daily 10-minute HR and SpO2 measures) in training data to predict death within 7 days [Demographic (Demo) + Vitals Train AUC]. The audience was asked to rank models based on how they thought the model would perform in real-world data (Audience Poll Rank). Finally, the moderator presented and ranked the results of the teams’ combined models’ performances in new data provided for testing (Demo + Vitals Test AUC, highlighted with bold font). Teams also developed baseline models using the same training and testing datasets but only variables known at birth to predict death before NICU discharge (Demo Train and Test AUCs).

## Discussion

**Fig. 2:**
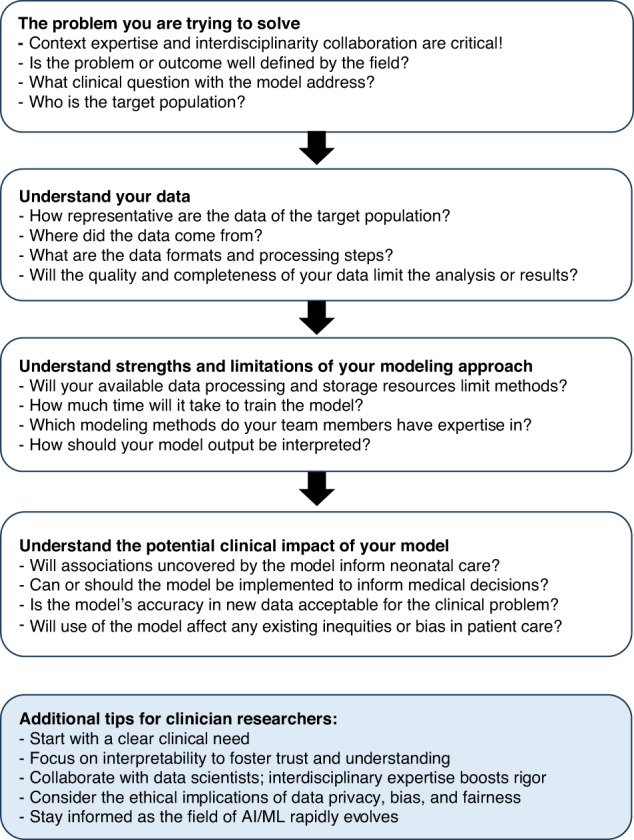
Considerations for using machine learning in neonatal research.

We conducted a modeling challenge to assess the performance of various ML approaches to predicting NICU mortality using a published dataset.^12^ This “bake-off” style competition provided valuable insights into the application of ML for predicting neonatal mortality. Contrary to the expectations of the audience and competitors, model complexity did not translate to superior “real world” performance. The logistic regression model’s success underscores the enduring relevance of ML using traditional statistical methods, particularly when combined with domain expertise. This finding challenges the prevailing enthusiasm for complex AI models, emphasizing that the optimal choice of methodology depends on multiple factors, including the research question, data characteristics, and the team’s expertise (Fig. 2). The following discussion highlights the key findings and implications for advancing research using ML models to predict neonatal outcomes.^4^

Our primary objective was to benchmark the performance of different ML methodologies using the same data and outcomes. In doing so, we identified heterogeneity in how researchers with variable technical and clinical expertise approached their assigned modeling task. Across the teams, we noted significant variability in the approach to tuning and evaluating model performance with the training data set. Results evaluated by the AUC in the test data set demonstrated that some models were more overfit than others, likely owing to the methods used to evaluate and tune performance with the training data. An “overfit” model is one that performs well with high discrimination on a training data set but has a significant performance drop when applied to novel data. Avoiding overfitting requires expertise with modeling in general as well as in the specific modeling methodology. Therefore, differences in model performance may not be the result of ML methodology itself, but rather their specific implementation. This serves as a cautionary tale for the development of any ML model; the final performance characteristics of a model when given unseen data (i.e., application to real world settings) depends on decisions made by the model developers.

Teams used different methods for handling variables with repeated measures, feature engineering, and tuning model parameters. The repeated vital signs measures from the same patients make the modeling process more vulnerable to overfitting. Team Wahoo accounted for the patient heterogeneity during model evaluation using a fast leave-one-cluster(patient)-out cross-validation,^13^ preventing the model from over-parameterization. The differences in approaches reflects the variability in options available for modeling and the experience of each team, which is likely even more significant when considering the rapid growth of ML in many different research fields, employed by interdisciplinary teams with a variable mix of clinical and technical backgrounds. For example, while logistic regression is a less complex modeling method, the team that used this method invested additional time and domain expertise to engineer new features, model non-linear relationships, and account for repeated measures to avoid overfitting. These results highlight the importance of evaluating not only the method of ML being used, but also the implementation decisions made by the researchers. It is crucial that researchers report sufficient detail for readers to understand and evaluate these details at a granular level and allow replication by others.

Before the modeling challenge, we needed to curate a common dataset large enough for multiple machine-learning approaches. The PhysioNet database hosted an international sepsis modeling competition using an open-source database with physiologic monitoring data from over 60,000 adult ICU patients at multiple hospitals.^14^ In contrast, our data set was curated from a single center with about 6,000 NICU patients for feasibility and focus, but multicenter data sets are preferred to test for generalizability. In similar methods as the PhysioNet challenge, we provided unseen test data that were not labeled with outcomes and waited to reveal the test data outcomes until after the model competition presentations. Ideally, any published model should be validated on external data or data not used in the training steps to evaluate the generalizability of the model’s performance. This is true for models intended for clinical implementation as well as those intended to test research hypotheses.

The results demonstrated that more complex models did not outperform simpler models for predicting NICU mortality across the various ML techniques, including traditional logistic regression, tree-based methods, and a neural network approach. Paradoxically, the logistic regression model had the highest AUC in the test data, while the audience voted it the least likely to perform well. These results demonstrate that excitement for complex modeling methods might be over-inflated, or at least that complexity is not correlated with predictive accuracy in all cases. In addition, the audience poll results indicate that attendees were influenced by the apparent virtues of a more complex methodology without knowing how well the model would perform on real-world data. This speaks to the potential for third-party AI tool vendors with good marketing techniques to generate excitement from clinicians over inadequately tested models.

Traditional methods have advantages when combined with domain expertise that should be used for benchmarking more complex modeling approaches. If the developers have captured all important associations in their input features, complex and simple models often perform similarly. As an example, two studies aiming to develop prediction models for early warning of late-onset sepsis evaluated multiple modeling methods on large data sets and found in both cases that logistic regression did just as well or better than the more complex methods.^15,16^

The under-performance of complex models in our competition highlights a critical concern in this rapidly developing field. While complex, cutting-edge techniques may offer the possibility of better predictive performance or faster computation times, they often come at the cost of less experience with optimization and calibration. It should also serve as a warning to medical consumers when evaluating commercial AI products, where good marketing techniques may highlight the sophistication of the technology to generate excitement at the expense of careful testing and calibration in real world settings. It is the opinion of the authors that all publication or marketing of machine learning models should include comparison to at least one simple model (e.g., logistic regression) to allow the reader to understand the improvement (or lack thereof) afforded by the more complex model.

The increased use of unsupervised ML models to predict clinical outcomes presents a similar double-edged sword. The unguided aspect of an unsupervised model may result in discovering novel and highly predictive patterns in the data; however, these models require careful human review to ensure novel associations are real and not an artifact of the training data (e.g., image AI detecting arrows marking the lesion as predictive). Additionally, interpretability remains a crucial aspect of deploying ML models in healthcare. Although advanced models like neural networks can provide superior performance in some situations, their “black-box” nature poses challenges for clinical interpretation and adoption. Fortunately, new tools are being developed to improve explainability of “black-box” models, such as the SHAP (SHapley Additive exPlanations) values^10^ used by the neural network model team. In contrast, simpler models inherently offer more intuitive insights into the relationship between predictors and outcomes, which may enhance clinician trust and facilitate integration into clinical decision-making processes.

An important result of this challenge was identifying key features that significantly contributed to the predictive models. Low gestational age, birth weight, Apgar scores, and heart rate variability were consistently among the top predictors across the different models. These findings align with existing clinical knowledge, which underscores the relevance of these features in neonatal mortality risk prediction. To address the interpretability of complex models, teams employed various methods to examine feature importance, which helped to demystify model predictions and explain feature contributions.^17^ Feature importance tools help to bridge the gap between model complexity and usability in a clinical context, especially in complex models where relationships are not readily apparent.

Using AUC as the sole metric to compare model performance limited the insights provided by the modeling challenge results. The ROC AUC is widely used in biomedical research due to its interpretability and ability to provide a single summary measure of a model’s discrimination ability across all possible thresholds. Additionally, it is threshold-independent, meaning it evaluates performance across all classification thresholds, making it robust against imbalances in class distribution. However, it has several limitations. First, it equally weights errors of omission (false negatives) and commission (false positives), which may not align with the clinical importance of one error type over another. Second, AUC does not evaluate goodness-of-fit, meaning it does not assess how well the predicted probabilities match the observed outcomes. A comprehensive model evaluation incorporating multiple metrics, though beyond the constraints of a conference presentation, should be performed for published models. These might include calibration, precision, recall, area under the precision-recall curve, sensitivity, specificity, F1 score, and positive/negative predictive values. Reporting multiple metrics enables more meaningful comparisons across models and provides a clearer understanding of model robustness and suitability for clinical application.

While logistic regression is conventional, reliable, and an interpretable model, particularly when enhanced by domain expertise, more advanced models like CatBoost, Random Forest, XGBoost, and neural networks have unique strengths that can lead to superior performance under specific circumstances. Here are several examples:

- CatBoost and XGBoost are highly effective models for working with categorical data, offering better performance with less feature engineering compared to logistic regression in many cases.^18,19^
- Neural networks perform particularly well when the data contains complex, multi-layered patterns, with many correlated or numerous variables that are hard to represent with linear models.^20^
- XGBoost handles modeling sparse data or datasets with missing values well. Thus, it can outperform simpler models in cases where built-in handling mechanisms for incomplete data are needed.^19^
- Random Forest can capture complex, non-linear relationships, works well with large datasets, is less sensitive to outliers and can automatically capture interactions between features.^21^
- Convolutional neural networks (CNNs) excel in processing image data, which is difficult to incorporate into traditional models.^22^

## Conclusions

In conclusion, our model challenge provided a hands-on opportunity for participants to explore the strengths and limitations of diverse modeling approaches, gain new insights into disease predictors, and engage in collaborative learning on emerging AI/ML techniques. The results underscore the importance of considering numerous factors when selecting an AI/ML methodology, which we outline in Fig. 2. With the growing complexity of medical datasets, hackathons and modeling challenges are proving to be invaluable for research teams eager to advance their skills and innovate. Figure 2 offers a roadmap for developing comprehensive ML models geared toward improving health outcomes.

Looking ahead, multidisciplinary collaboration and validation across large, multicenter datasets will drive progress toward more robust, clinically relevant algorithms. This modeling challenge not only serves as a guide for future researchers but also highlights the potential of collaborative research to shape impactful, real-world solutions in healthcare. With combined clinical and data science expertise, we can look forward to transformative advancements in predictive modeling that will meaningfully enhance patient care.

## Data Availability

The dataset used for this competition is available online at 10.18130/V3/5UYB4U (see reference #6).
